# Measuring the effectiveness of the Covenant of Mayors on the reporting of climate hazards by Municipalities

**DOI:** 10.1016/j.heliyon.2020.e05043

**Published:** 2020-10-06

**Authors:** Yeray Hernandez, Gustavo Naumann, Paulo Barbosa

**Affiliations:** European Commission, Joint Research Centre, Ispra, Italy

**Keywords:** Climate change, Hazards, Agreement, Covenant of mayors, Environmental science, Climatology, Climate policy, Earth sciences, Natural hazard

## Abstract

The European Commission established the Covenant of Mayors (CoM) initiative in 2008, aimed at involving and supporting mayors to encourage accomplishing the European Union (EU) climate mitigation and energy targets. In 2014, the Mayors Adapt initiative was set up in order to promote the climate adaptation pillar. Whereas the mitigation pillar is more developed and peer-reviewed literature can be found, adaptation is still lagging behind, not to mention the absence of information on the effectiveness of the CoM concerning the development of climate adaptation plans. This paper aims at presenting a thorough analysis of climate hazard data declared by CoM signatories as well as the degree of regional agreement of those signatories when reporting climate data. Thus, we assume that the signatories belonging to the same climate region should report similar climate hazard data for both current and future timeframes. Using a new statistical method for measuring the variability of categorical data, we determine that, overall, the signatories show low agreement within climate regions. Hence, we conclude that the CoM, in the corresponding part of climate risk assessment, is not as effective as it could be desired. Furthermore, several recommendations are proposed to improve the current reporting.

## 1 Introduction

The Covenant of Mayors (CoM) initiative was established by the European Commission in 2008, aimed at involving and supporting mayors to encourage accomplishing the European Union (EU) climate mitigation and energy targets (CoM, 2019). In 2014, the Mayors Adapt initiative was set up in order to promote the climate adaptation pillar. Mayors Adapt encouraged local governments to support the development and implementation of climate adaptation actions. In 2015, the mitigation and adaptation pillars were merged to support the implementation of the EU 40% GHG-reduction target by 2030, adopting an integrated approach to climate change mitigation and adaptation, and ensuring access to secure, sustainable and affordable energy for all. In 2016, the CoM joined forces with the Compact of Mayors initiative, resulting in the Global Covenant of Mayors for Climate and Energy (GCoM, 2019). The GCoM has already involved over 10,000 signatories, covering around 800 million inhabitants (GCoM, 2019).

With the aim of guaranteeing that the adaptation pillar of the CoM initiative lines up with its codes, the Joint Research Centre, develops an evaluation of the action plans submitted by the Municipalities, including the Risk and Vulnerability Assessments. The evaluation follows a set of eligibility criteria that must be fulfilled in order to have the action plan accepted (Barbosa et al., 2018). If the signatories are compliant with all the mandatory criteria, their submission is accepted and they will receive a positive feedback report; otherwise, they will receive a feedback report with recommendations for improving the action plan and re-submit the information. Furthermore, the non-compliant signatories usually are encouraged to engage with universities, research groups or other key stakeholders.

There is not much literature available on the CoM, especially on the adaptation component of the initiative. One of the first publications that can be found is the analysis carried out by Christoforidis et al. (2013) to investigate the situation of the CoM in Greece. The authors concluded that the main barriers to the full implementation of the initiative is the lack of information and communication to the Greek citizens; they were neither aware of their mayors' signing nor about the taken commitments. With regards to climate risk assessment, Aguiar et al. (2018) examined 147 local adaptation strategies in Europe concluding that flooding and droughts are the most urgent hazards to be addressed in Europe; the lack of resources, technical capacity, political commitment, as well as the uncertainties were the main barriers identified in this study. These authors also concluded that the inclusion of social actors and citizens in climate planning could enhance the identification of local and regional vulnerabilities (Aguiar et al., 2018).

Regarding adaptation actions, Geneletti and Zardo (2016) developed a framework to analyse the treatment of ecosystem-based actions in CoM signatories, indicating the lack of specific information and poor implementation to reduce population vulnerability. Pasimeni et al. (2019) analysed the synergy between mitigation and adaptation actions using CoM signatories' information. The authors indicate that Italian large and medium-sized signatories tend to develop actions to tackle simultaneously mitigation and adaptation. Lastly, Melica et al. (2018) addressed the multi-level model of climate governance, called Covenant Territorial Coordinators (CTCs), to showcase how CTCs may help small local authorities to carry out climate actions plans within the CoM by means of financial and technical support.

In this context, the present paper is therefore important since it is the first time, to the authors' knowledge, a thorough analysis of the climate hazard data and its degree of regional agreement is carried out. A high level of agreement happens when the Municipalities of the CoM belonging to the same climate region report a similar climate hazard both in present and future times. On the other hand, a low level of agreement happens when there is a high level of variability of the climate hazard inside the same climate region. The results obtained could highlight the effectiveness of the climate adaptation component of the CoM.

Assuming that Municipalities located in the same climate regions should be threatened by similar climate hazards in present and future, our research question that will be analysed and discussed in this paper is the following: *are the signatories of the CoM that belong to the same climate region reporting similar levels of a particular climate hazard data for both present and future?* This study attempts to provide reasons for the possible differences within similar climate regions as well as solutions to improve the quality and effectiveness of the reporting.

## 2 Material and methods

### 2.1 Material

All the 137 Municipalities that had submitted their climate risk assessment to the MyCovenant platform (CoM, 2019), by November 2018, were included in this analysis. These 137 Municipalities were asked to report the weather and climate hazards that are relevant for them for both the present and the future (see description in Tables 1 and 2). Furthermore, they were also asked to indicate the expected change in intensity and frequency of the hazard, as well as the timeframe in which those changes are expected to happen (see Table 1 as an example).

**Table 1:** Dropdown list as found in the MyCovenant Platform.

Current hazard *risk* level	Anticipated *risks*		
	Expected change in intensity	Expected change in frequency	Timeframe
Low	Increase	Increase	Current (nowadays)
Medium	Decrease	Decrease	Short-term (0-5 years)
High	No change	No change	Medium-term (5-15 years)
Not known	Not known	Not known	Long-term (over 15 years)
			Not known

There is a misuse of the concepts “hazard” and “risk” in the *MyCovenant* platform (see *italics* in the Table). *MyCovenant* platform refers to current hazard risk level when it should be “current hazard level”, mixing definitions of hazard and risk (see Table 7). Hazards are only a component of risk, along with exposure and vulnerability. Similarly, *MyCovenant* platform refers to the expected change in intensity and frequency as “anticipated risks”, when it should be “anticipated hazards” since the risk cannot change in intensity or frequency (only the hazard can).

Source: CoMO (2016).

**Table 2:** Definitions of climate hazards as found in the CoM platform.

Hazard	Description
Extreme heat	Temperature being above the 90^th^ percentile of the daily maximum temperature.
Extreme cold	Temperature being below the 10^th^ percentile of minimum temperature.
Extreme precipitation	Not defined.
Floods	The overflowing of the normal confines of a stream or other body of water, or the accumulation of water over areas that are not normally submerged. Floods include river/fluvial floods, flash floods, pluvial floods, sewer floods, coastal floods, etc.
Sea level rise	Not defined.
Droughts	A period of abnormally dry weather long enough to cause a serious hydrological imbalance.
Storms	An atmospheric disturbance that can be manifested in strong winds accompanied by rain, snow, or other precipitation and by thunder and lightning.
Landslides	A mass of material that has moved downhill by gravity, often assisted by water when the material is saturated. The movement of soil, rock, or debris down a slope can occur rapidly, or may involve slow, gradual failure.
Forest fires	Not defined.

Source: MyCovenant (CoM, 2019).

From the list of hazards indicated in Table 2, we concentrated on extreme heat, extreme cold, floods, sea level rise, droughts, and storms. This allows focusing only on the most relevant hazards reported by the municipalities. Furthermore, for this analysis we have included the “extreme precipitation” hazard in the floods hazard type.

### 2.2 Environmental-climate stratification

**Figure 1:**
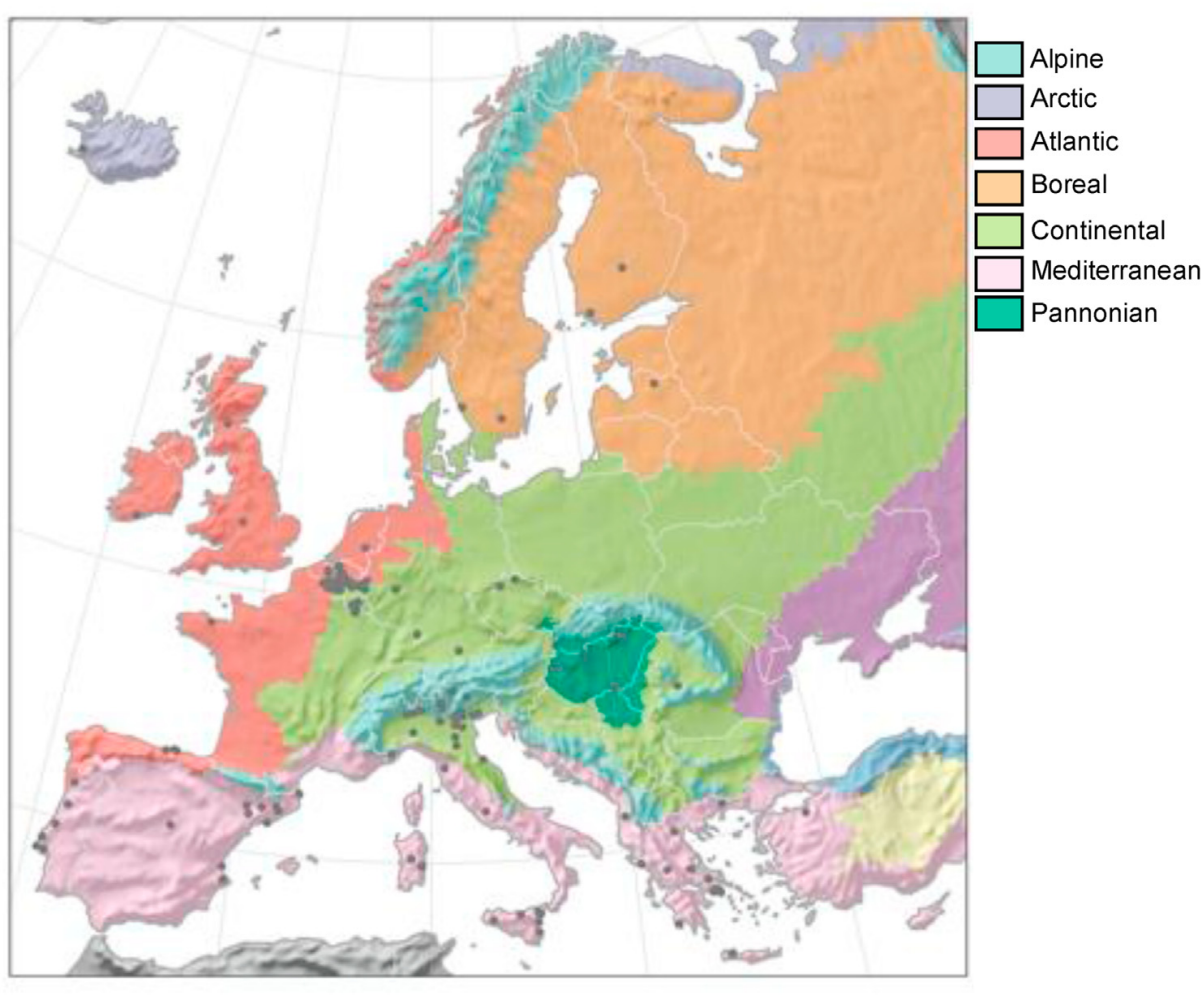
Geographical location of the 137 CoM municipalities considered in the analysis and bioregions to which they belong.

Climate classifications are usually based on threshold values of seasonal monthly air temperature and precipitation. Over same climate regions similar biomes are mapped, i.e. different regions in a similar class share common climate and vegetation characteristics. The regional classification used here is based on the environmental stratification in bioregions proposed by Metzger et al. (2005). This dataset distinguishes 17 different zones globally. However, the municipalities available were located only in seven main bioregions. These bioregions are: Mediterranean (MED), Atlantic (ATL), Continental (CON), Alpine (ALP) Pannonian (PAN), Boreal (BOR), and Arctic (ARC). That means that the main spatial climatic features are similar, except for some local conditions that are influenced by orography, proximity to the sea, or in transition zones. According to these definitions, we assume that climatic hazards (and thus Municipalities reports) should behave similarly within the same zone while still allowing small variations due to the aforementioned local effects. In order to answer the research questions, the CoM municipalities were grouped in these different climate regions and their statistical variability was analysed in each of them (Figure 1). It is worth noting that only four municipalities belong to the Boreal region while only one belongs to the Arctic region. To avoid small sample biases, we do not include these municipalities in our analysis, however, their statistical estimates are incorporated in Tables 4 and 5.

### 2.3 Statistical method

As presented in Table 3, the municipalities signatories of the CoM, were asked to report the present hazard level (low, medium, high, or not known) per type of hazard (droughts, extreme heat, extreme cold, extreme precipitation, floods, sea level rise, and storms), as well as what changes they expect in the future in terms of intensity, frequency and time frame. These relative changes were categorized in four classes (not known, decrease, no change, and increase) while the time frame for the changes was classified as current/short term (0–5 years ahead the baseline), medium term (5–15 years) and long term (more than 15 years). This information provided by the Municipalities is represented by different ordered categorical variables as they are placed into different categories according to different levels of intensity or rate of change. A new category, called “Not mentioned”, was added in case the signatories did not mention a climate hazard, while other signatories in the same cluster mention it.

**Table 3:** Nomenclature used for reported hazards levels’ and future frequency and intensity.

Current hazard	Future		
	Intensity	Frequency	Time frame (∗)
Low	Increase	Increase	Current/Short term∗∗
Medium	No change	No change	Medium term
High	Decrease	Decrease	Long term
Not known	Not known	Not known	Not known
(Not mentioned)	(Not mentioned)	(Not mentioned)	(Not mentioned)∗∗∗

(∗) refers to the period of time when the Municipality expects the hazard to change in terms of either intensity of frequency (or both). The timeframe that can be chosen is the following: current (at the time of the survey), short-term (0–5 years), medium-term (5–15 years), long-term (over 15 years) or not known (CoMO, 2016). (∗∗) for the purpose of this analysis current and short term were merged (CoMO, 2016). (∗∗∗) A “not mentioned” category was added in case the signatories did not mention a climate hazard, while other signatories in the same cluster mention it.

As mentioned in the previous sections, the data introduced by the municipalities were clustered in terms of bioregions (see Figure 1). Thus, each bioregion has associated a table of frequencies indicating the number of times a present hazard has been flagged as low, medium, high, not known, or was not mentioned by the Municipality. Intuitively, if one category has 100% frequency, the variability within the cluster becomes zero; this would highlight that the cluster/bioregion shows a high agreement (e.g. they have probably used the same climate models and/or have been assisted by a CTC). However, if all the five categories have the same frequency then the variability is maximised. In this case, the reported data for the climate hazard considered in the bioregion shows a low agreement and the municipalities are not using the same climate models (in case they are doing so) and/or are not assisted by any CTC (other possible causes are discussed in section 4).

The notion of variability and similarity for categorical data is not as straightforward as for continuous data. For instance, arithmetic operations cannot be done directly in categorical variables. In order to represent the maximum and minimum variability, we adopt the method proposed by Allaj (2017), who applied the method to an example of customer satisfaction of mobile companies. This statistical method is useful to analyse the variability of categorical data. Notably, the method proposed by Allaj (2017) has been applied to diverse case studies. For example, to calculate the inter-annual variability of flood generating processes (Stein et al., 2020) as well as to medical case studies. Aquino et al. (2018) applied the variability index $v_{k,s}$ to investigate the reliability of Foot Posture Index of foot posture of adults and older adults. Shiffman et al. (2018) also applied the variability index in order to develop a model for probabilistically reconstructing trees of cellular differentiation. Bilz et al. (2019) used the variability index to « reveal a synaptic rather than cellular distribution of a memory code and coherence of distributed synaptic activity as a memory-encoding parameter». Thus, the variability of an outcome resulting from categorical data with *n* elements falling in *k* categories 0, 1, 2, …, *k* – 1 with vector of the relative frequencies equal to **f** = (*f*_0_, *f*_1_, …, *f*_k-1_) is restrained by the following variability indexes:

$$v_{k}=1-{\left\|f\right\|}_{k}=1-\sqrt{f_{0}^{2}+f_{1}^{2}+\ldots +f_{k-1}^{2}}$$

$$v_{k,s}=\frac{v_{k}}{1-\frac{1}{\sqrt{k}}}$$

where $f_{i}=\frac{n_{i}}{n}$, for any *i* = 0, 1, 2, …, *k* – 1, where *n*_*i*_ provides the number of elements falling in category *i.* $n=\;\overset{k-1}{\underset{i=0}{\sum }}n_{i}\geq 1$ is the relative frequency of the *i*-th category and || · ||_*k*_ is the Euclidean norm defined on the real space R^*k*^. $v_{k}$ maximum variability equals approximately 0.533. Therefore, $v_{k,s}$ is used to standardise the variability of the categories between the values zero and one. When this variability index equals zero, the *n* elements fall within a single category (as indicated above), and when the variability index makes $1-\frac{1}{\sqrt{k}}$ or 1, the *n* elements are equally distributed across the different categories.

## 3 Results

The results of the analysis are shown in the following three subsections. We start presenting the input data without any statistical treatment, i.e. the raw material as found in the *MyCovenant* platform. Thus, we present the main trends in the reporting of the climate hazard data by the Municipalities under analysis. Secondly, in sections 3.2 and 3.3, the statistical method is applied to the input data and the level of (dis)agreement is presented in detail per bioregion. In this section, to avoid small sample biases, only bioregions with more than 5 reporting municipalities were analysed, i.e. Mediterranean (MED), Atlantic (ATL), Continental (CON), Alpine (ALP) and Pannonian (PAN).

### 3.1 Main trends for climate hazards

**Figure 2:**
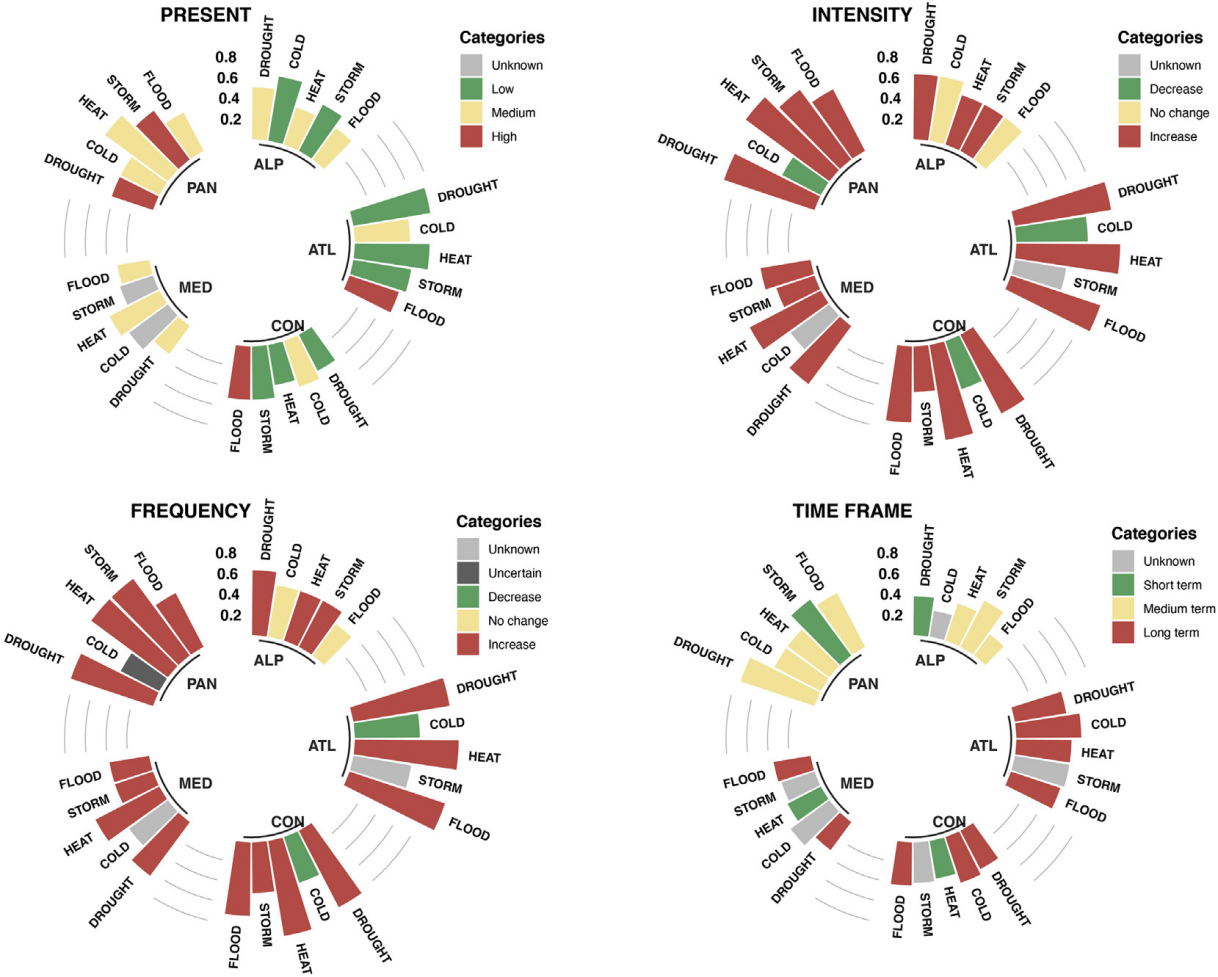
Top reported categories by the municipalities of current hazard level, expected changes in future intensity, frequency, and time frame (as from Table 3) per hazard and bioregion. The colours represent the most reported category while the values in the vertical axis are expressed in terms of relative frequency indicating the times the most selected category has been flagged in each bioregion (see Table 4). The results are presented for the five bioregions: Mediterranean (MED), Continental (CON), Atlantic (ATL), Pannonian (PAN) and Alpine (ALP) and five hazards: drought, cold and heat waves, storm and floods. Uncertain (dark grey) refers to a unique case where half of signatories reported increase, whereas the other half reported decrease.

Figure 2 shows the predominant frequencies (i.e. the category with the highest count) indicating the number of times a present hazard has been flagged in the most selected category (low, medium, high, not known, or was not mentioned) in each cluster. The same information is presented also for the expected changes in the future in terms of intensity, frequency and time frame.

For the present climate conditions, most of the clusters agree with the hazard categories in line with the regional predominant climate. According to this information, all hazards were reported as a present threat over all regions with the exception of cold waves and storms that were mostly not mentioned in the Mediterranean region.

Floods are the only hazard considered as a medium to high threat in all the regions. As this hazard type includes all kinds of floods (river/fluvial floods, flash floods, pluvial floods, sewer floods, coastal floods) most of the Municipalities are affected by any of these events. The natural effects of flooding are usually aggravated by changes in urban landscapes that alter the hydrological nature of surface runoffs and restrict infiltration of surface water, producing, under some conditions, the water moves to rivers faster than it does under natural conditions. In terms of impacts, floods were highlighted as one of the major causes of economic and human losses in urban centres in Europe (Alfieri et al., 2016).

Drought is a climate hazard that can occur in almost all climates and its onset is due to a temporary reduction of precipitation that can be further exacerbated by high temperatures. Semi-arid regions, like the Mediterranean, are more susceptible to the effects of droughts as usually the water available barely outbalances the demand. Consistently with recent reported events, droughts are considered as a medium to high threat in the ALP, PAN and MED and considered a low threat in the ATL and CON where more water availability could hide the effects of short droughts. On the other side, drought is mostly considered as a medium threat in MED while a high threat would have been expected due to future climatic conditions.

Even though, heat waves are associates to large scale meteorological systems, the effect of heat extremes can be further exacerbated in cities as dense urban areas are often significantly warmer than the surrounding countryside (Ward et al., 2016). The air temperature in dense cities can be 5–10 °C warmer compared to the neighbouring rural areas. This is known as the urban heat island effect, and worsens the effect of heat waves, mainly during the night. According to the reported data, heat waves are considered as a medium threat in the MED, PAN, and ALP but surprisingly, are only considered as a low threat in ATL and CON, maybe because in some regions longer summers with warmer temperatures could be perceived as beneficial.

There is an inverse relation in the reported data of both temperature extremes: the clusters that declared cold extremes as medium, reported the extreme hot events as low and vice-versa. The only region that reported both extremes as a threat is PAN, consistent with the definition of continental climates often characterized by very hot summers and extreme cold winters. In that way, cold extremes are considered as medium threat in the ATL, CON and PAN while in the Mediterranean, almost half of the municipalities did not mention cold waves as a relevant hazard.

Regarding the future conditions, there is an overall good agreement between intensity and frequency where most of the cities (usually >50%) in all clusters declares an increase in both variables for all hazards. The main exception is cold waves where intensity and frequency are expected to decrease in the ATL and CON regions.

Particularly, cold extremes are the only hazard which presents many disparities across clusters and climatic feature (intensity or frequency). In the Alpine region no changes are expected while in the PAN region a decrease is declared for the intensity but no agreement was observed for the frequency where the same number of municipalities declared increase and decrease. Only in the MED region is not mentioned; however, this is consistent with the low relevance declared for the present conditions.

In terms of time frame, also seems to be a good agreement per cluster/region but not between them. In PAN and ALP most of the effects are expected to be felt in the medium term while in ATL and CON the long term is the most selected time frame. In the MED region heat waves are reported for the short term, while drought and floods are expected in the long term. No agreement in the time frame is observed for cold and storm in this region.

The municipalities report that drought frequency and intensity will increase in all European regions. According to many recent studies that use regional climate and hydrological models this is true for the MED and ATL regions (Marx et al., 2018; Spinoni et al., 2018; Samaniego et al., 2018); however, droughts change in the PAN and CON regions are more uncertain and the robustness in the sign of change depend on the indicator used to define drought conditions (Trenberth et al., 2014).

**Table 4:** Number of cities per bioregion and top reported categories and their relative frequency of current hazard level, expected changes in future intensity, frequency and time frame per hazard and bioregion.

Hazard	Cluster	Number of cities	Label	Freq.	Label	Freq.	Label	Freq.	Label	Freq.
Droughts	ALP	8	Medium	0.50	Increase	0.63	Increase	0.63	Short term/Current	0.38
	ARC	1	NA	NA	NA	NA	NA	NA	NA	NA
	ATL	32	Low	0.75	Increase	0.94	Increase	0.94	Long term	0.50
	BOR	4	Medium	0.50	Increase	1.00	Increase	0.75	Long term	0.50
	CON	41	Low	0.39	Increase	0.85	Increase	0.83	Long term	0.41
	MED	39	Medium	0.33	Increase	0.69	Increase	0.64	Long term	0.33
	PAN	12	High	0.42	Increase	0.92	Increase	0.83	Medium term	0.75
Extreme cold	ALP	8	Low	0.63	No change	0.63	No change	0.50	Not Known	0.50
	ARC	1	NA	NA	NA	NA	NA	NA	NA	NA
	ATL	32	Medium	0.53	Decrease	0.69	Decrease	0.63	Long term	0.63
	BOR	4	Low	1.00	Not known	0.50	Decrease	0.50	Long term	0.75
	CON	41	Medium	0.46	Decrease	0.49	Decrease	0.46	Long term	0.46
	MED	39	Not mentioned	0.46	Not mentioned	0.46	Not mentioned	0.46	Not mentioned	0.46
	PAN	12	Medium	0.42	Decrease	0.42	Decrease	0.42	Medium term	0.50
Extreme heat	ALP	8	Medium	0.38	Increase	0.50	Increase	0.50	Medium term	0.38
	ARC	1	NA	NA	NA	NA	NA	NA	NA	NA
	ATL	32	Low	0.72	Increase	1.00	Increase	1.00	Long term	0.53
	BOR	4	Low	0.75	Increase	1.00		1.00	Long term	0.75
	CON	41	Low	0.39	Increase	0.93	Increase	0.93	Short term/Current	0.37
	MED	39	Medium	0.54	Increase	0.77	Increase	0.69	Short term/Current	0.36
	PAN	12	Medium	0.75	Increase	1.00	Increase	0.92	Medium term	0.50
Storms	ALP	8	Low	0.50	Increase	0.50	Increase	0.50	Medium term	0.50
	ARC	1	NA	NA	NA	NA	NA	NA	NA	NA
	ATL	32	Low	0.56	Not known	0.50	Not known	0.56	Not known	0.53
	BOR	4	High	0.25	Increase	0.75	Increase	0.75	Medium term	0.50
	CON	41	Low	0.51	Increase	0.44	Increase	0.49	Not known	0.39
	MED	39	Not mentioned	0.33	Increase	0.38	Increase	0.38	Not mentioned	0.33
	PAN	12	High	0.58	Increase	0.83	Increase	0.92	Short term/Current	0.67
Sea level rise	ATL∗	14	Medium	0.36	Increase	0.82	Increase	0.82	Medium term	0.64
	MED∗∗	20	Low	0.61	Increase	0.61	Increase	0.56	Long term	0.50
Floods	ALP	8	Medium	0.38	No change	0.50	No change	0.38	Medium term	0.25
	ARC	1	NA	NA	NA	NA	NA	NA	NA	NA
	ATL	32	High	0.50	Increase	0.91	Increase	0.97	Long term	0.50
	BOR	4	Medium	0.50	Increase	1.00	Increase	1.00	Short term/Current	0.50
	CON	41	High	0.51	Increase	0.73	Increase	0.71	Long term	0.41
	MED	39	Medium	0.31	Increase	0.49	Increase	0.38	Long term	0.36
	PAN	12	Medium	0.42	Increase	0.67	Increase	0.58	Medium term	0.58

∗Includes one Municipality from BOR and one from CON. ∗∗ Includes one Municipality from CON. NA not applicable.

### 3.2 Degree of agreement of climate hazard data

**Figure 3:**
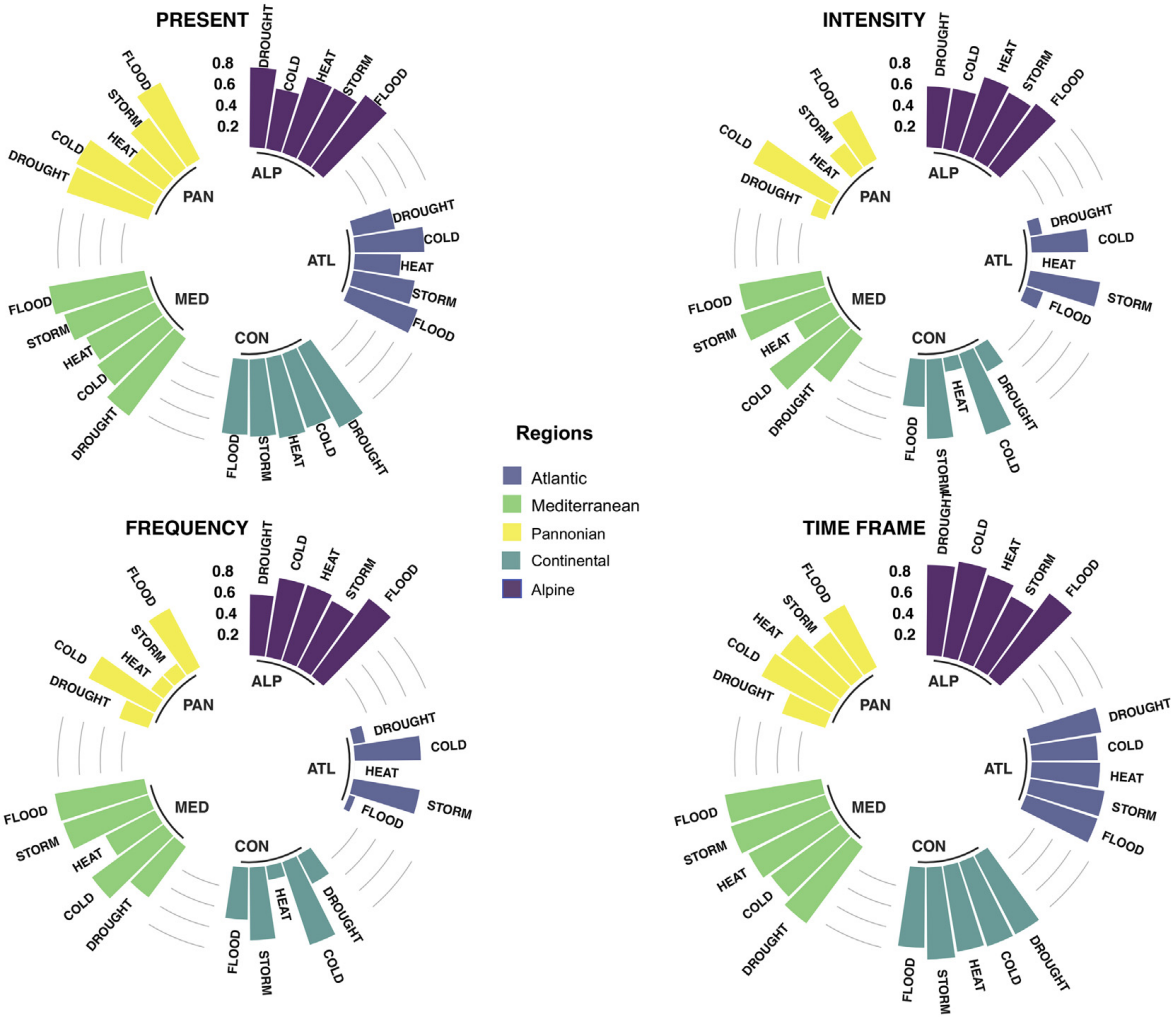
Degree of regional agreement ($v_{k,s}$) for reported categories by the municipalities of present hazard level, expected changes in future intensity, frequency and time frame per hazard and bioregion (see Table 5). The results are presented for the five bioregions: Mediterranean (MED), Continental (CON), Atlantic (ATL), Pannonian (PAN) and Alpine (ALP) and five hazards: drought, cold and heat waves, storm and floods. For $v_{k,s}$ = 0 the n reports of each region are within a single category (full agreement), while when $v_{k,s}$ = 1 the n elements are equally distributed across the different categories (low agreement).

A summary of the results can be seen in Figure 3. Four charts are presented to visualise the degree of agreement of the clusters per type of climate hazard. The main results are fourfold, i.e. present time (how the Municipalities expect climate hazards to be in the present), and how they expect them to be in the future, in terms of intensity (how hard they will strike), frequency (how many times they will happen), and time frame (when). In the following paragraphs, the main results will be described.

In general, the reported data shows a low level of agreement. As indicated in section 2.3, a cluster shows high agreement if the variability of the categories tends to zero (as said, there could be many explanations to why slight differences may occur in terms of hazards in the same cluster). Instead, if it tends to one, the cluster presents low agreement, indicating that something is potentially wrong in the data provided by the Municipalities.

Thus, complete disagreement is found in few cases (clusters ATL and PAN for intensity, and ATL for frequency), all of them linked to future extreme heat. High agreement ($v_{k,s}$ <0.25) is found for future projections, this time linked to extreme heat, but also droughts, floods, and storms. The cluster ATL appears to be the one presenting high agreement, although CON presents remarkable low values for extreme heat (intensity and frequency), whereas PAN performs well for droughts (intensity), extreme heat (frequency), and storms (frequency). On the other hand, low agreement ($v_{k,s}$ >0.75) is found in more cases, for both present and future. Starting from the present, all cluster but ATL indicates low agreement, at least, for one hazard: MED (floods, droughts, storms, and extreme cold), PAN (extreme cold, droughts, and floods), CON (droughts and extreme heat), and ALP (floods). Regarding the intensity expected for the future, low agreement is found for MED (storms and extreme cold), PAN (extreme cold), and CON (extreme cold). In the case of frequency, MED presents again low agreement (floods, storms, and extreme cold), ALP (floods), and CON (extreme cold). Lastly, the lowest agreement is found in the timeframe, concretely for MED (all hazards), CON (all hazards), and ALP (all hazards but storms).

**Table 5:** Degree of regional agreement ($v_{k,s}$) for reported categories of present hazard level, expected changes in future intensity, frequency and time frame per hazard and bioregion.

Hazard	Cluster	Number of cities	Present	Future		
				Intensity	Frequency	Time frame
Droughts	ALP	8	0.75	0.57	0.57	0.85
	ARC	1	NA	NA	NA	NA
	ATL	32	0.40	0.11	0.11	0.68
	BOR	4	0.53	0.00	0.38	0.70
	CON	41	0.85	0.25	0.30	0.83
	MED	39	0.90	0.51	0.59	0.89
	PAN	12	0.81	0.14	0.29	0.43
Extreme cold	ALP	8	0.57	0.57	0.75	0.91
	ARC	1	NA	NA	NA	NA
	ATL	32	0.65	0.53	0.62	0.62
	BOR	4	0.00	0.70	0.70	0.38
	CON	41	0.73	0.80	0.82	0.84
	MED	39	0.77	0.83	0.84	0.81
	PAN	12	0.83	0.83	0.70	0.74
Extreme heat	ALP	8	0.75	0.75	0.75	0.85
	ARC	1	NA	NA	NA	NA
	ATL	32	0.43	0.00	0.00	0.64
	BOR	4	0.38	0.00	0.00	0.38
	CON	41	0.76	0.13	0.13	0.82
	MED	39	0.72	0.40	0.52	0.88
	PAN	12	0.41	0.00	0.14	0.70
Storms	ALP	8	0.75	0.70	0.70	0.75
	ARC	1	NA	NA	NA	NA
	ATL	32	0.58	0.66	0.63	0.71
	BOR	4	0.90	0.38	0.38	0.70
	CON	41	0.73	0.75	0.68	0.86
	MED	39	0.83	0.84	0.84	0.94
	PAN	12	0.58	0.29	0.14	0.51
Sea level rise	ATL∗	14	0.90	0.29	0.28	0.67
	MED∗∗	20	0.74	0.63	0.73	0.86
Floods	ALP	8	0.85	0.75	0.91	0.96
	ARC	1	NA	NA	NA	NA
	ATL	32	0.68	0.17	0.06	0.70
	BOR	4	0.70	0.00	0.00	0.70
	CON	41	0.71	0.45	0.49	0.75
	MED	39	0.91	0.78	0.85	0.92
	PAN	12	0.81	0.51	0.62	0.66

∗ Includes one Municipality from BOR and one from CON. ∗∗ Includes one Municipality from CON. NA not applicable.

Even though, present data should intuitively indicate higher agreement than the future data, the results show contradictory information. The time frame presents lower agreement (minimum of $v_{k,s}$ = 0.427 for droughts in PAN and maximum of 0.963 for floods in ALP) than present times (0.395 for droughts in ATL and maximum of 0.908 for floods in MED) –something that in principle makes sense due to the inherent uncertainty associated to simulating the future climate–. However, intensity and frequency (referring to the future climate) show the opposite, i.e. long-term appears to show more agreement than nowadays, at least, for extreme heat, droughts, floods, and storms (this latter in the case of PAN). This anomalous result and contradictory information will be discussed in the following section.

In terms of future data, both, intensity and frequency show similar results. Firstly, same level of agreement between intensity and frequency is found for ALP (droughts, extreme heat, and storms), ATL (droughts and extreme heat), and CON (extreme heat). However, frequency presents slightly less agreement than intensity. Secondly, intensity presents low agreement for MED (storms and extreme cold), PAN (extreme cold), and CON (extreme cold), whilst frequency shows low agreement in MED (floods, storms, and extreme cold), ALP (floods), and CON (extreme cold), i.e. one more hazard (floods).

Extreme heat is the hazard that shows the highest agreement. However, not all clusters indicate the same pattern. In the present, extreme heat is the best performer, excluding the clusters ALP (extreme cold), ATL (droughts), and CON (storms). In the case of intensity, extreme heat is again the best performer, excluding ALP (droughts and extreme cold). Concerning frequency, extreme heat stands out again excluding ALP (droughts). However, in the time frame, extreme heat is never the best performer.

On the other hand, extreme cold and floods indicates low agreement. In the present, floods present the lowest agreement for all clusters but CON (droughts) and PAN (extreme cold). Instead, extreme cold shows the lowest agreement for the future intensity in CON and PAN, as well as for frequency. Lastly, floods make the lowest agreement in the time frame for ALP and MED.

### 3.3 The special case of sea level rise

**Figure 4:**
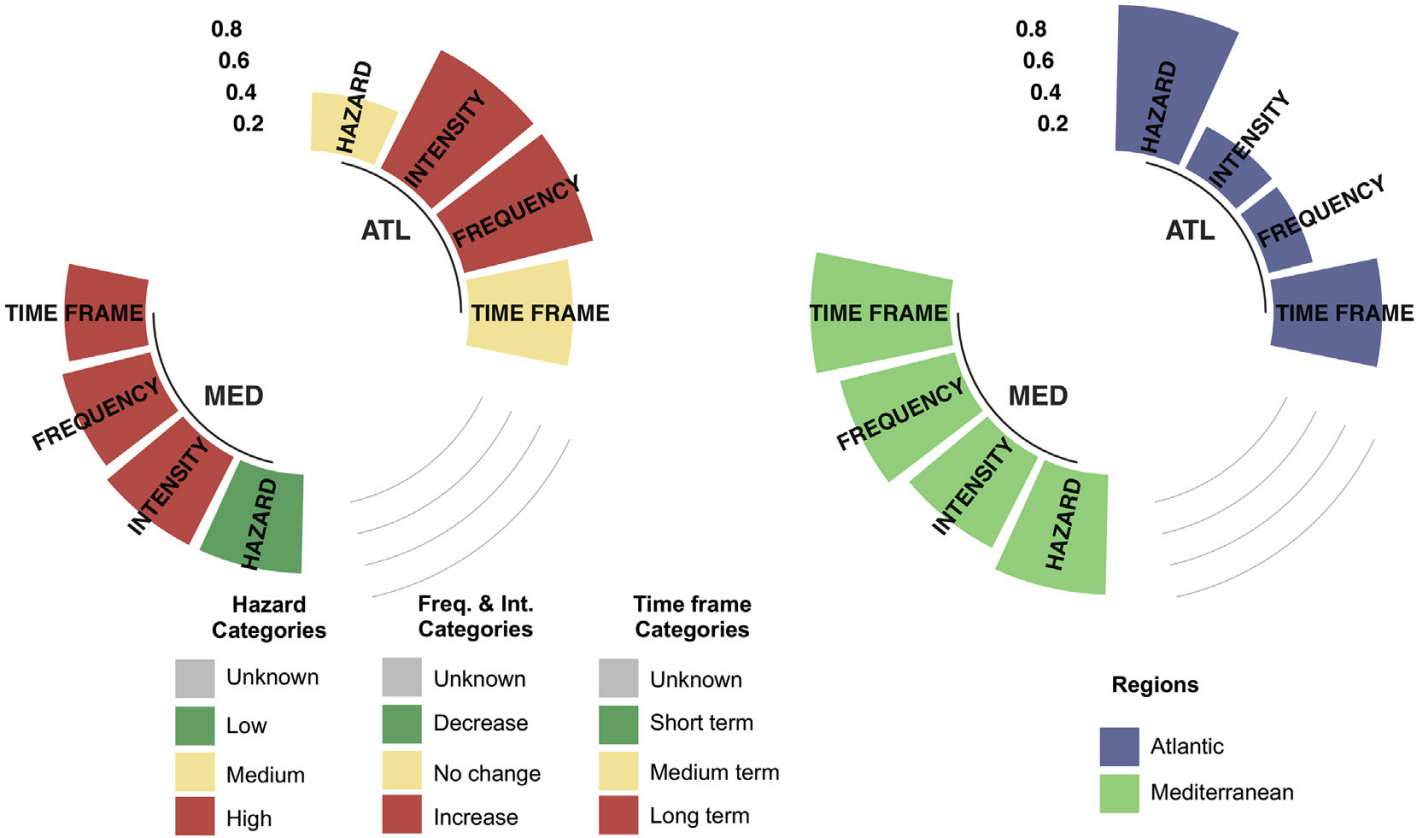
Top reported categories by the municipalities (left) and Degree of regional agreement ($v_{k,s}$) for reported categories by the municipalities of present Sea Level rise level, expected changes in future intensity, frequency and time frame of Sea Level Rise over the relevant Mediterranean and Atlantic regions. Values in the vertical axis in the left panel are expressed in terms of relative frequency indicating the times a present hazard has been flagged in the most selected category in each bioregion (Table 4) while the vertical axis in the right panel represents the degree of agreement between regions ($v_{k,s}$, see Table 5). Only municipalities located on coastal zones in the Atlantic and Mediterranean regions are included.

Sea level rise has been analysed separately, as the spatial extent of this hazard is confined to the coastal zones, thus affecting only municipalities located by the sea side. Consequently, the Municipalities have been split up into two clusters using the reporting data for sea level rise. The clusters are the following: those located in the Atlantic Ocean (called ATL) and those located in the Mediterranean Ocean (called MED). The results are shown in Figure 4.

For the present climate conditions, sea level rise is considered mostly a low hazard in the Mediterranean and a medium threat in the Atlantic. Regarding the future conditions, the majority of the Municipalities (more than 50% in MED and around 80% in ATL) declared an increase in intensity and frequency. These changes are projected to be felt in the medium term in the ATL and in the long term in MED.

In general, the agreement tends to be low. First of all, complete agreement has not been found, as well as high agreement ($v_{k,s}$ <0.25) in this special case. On the other hand, low agreement ($v_{k,s}$ >0.75) is found for both clusters: for the present in the case of ATL and time frame in the case of MED.

Counterintuitively, present data indicates a lower agreement compared to future projections. Indeed, the degree of agreement of both clusters are rather low for the present ($v_{k,s}$ = 0.901 for ATL and 0.741 for MED). For example, the degree of agreement of future intensity is somewhat medium ($v_{k,s}$ = 0.294 for ATL and 0.632 for MED); the degree of agreement of future frequency is also medium ($v_{k,s}$ = 0.28 for ATL and 0.732 for MED); and the degree of agreement of the time frame is rather low ($v_{k,s}$ = 0.668 for ATL and 0.861 for MED).

Although Municipalities are required to report intensity and frequency of sea level rise, it does not make sense to define its intensity and frequency, since this is a slow increasing process of sea volume due to increasing sea temperature and to ice melting.

## 4 Discussion

In this section, the results pointed out above are discussed, including possible causes for the low agreement and concrete actions that could be taken to increase the degree of agreement within each climate region, by improving the reporting platform and process.

Regarding the research question *“are the Municipalities signatories of the CoM, belonging to the same climate region, reporting similar climate hazards data for both present and future?”*, the answer is no, although there are some differences between current and future hazards. As indicated above, the variability should tend to zero as an indication of high agreement within climate regions. For the present, in general, there is less agreement (higher variability) than for the future in terms of intensity and frequency.

According to our analysis, the low agreement found within climate regions shows that the reporting platform would benefit from a better definition of concepts and questions. A consistent climate hazard assessment is fundamental to develop risk assessments that will inform the climate adaptation plans, since the adaptation actions proposed by the Municipalities (should) emerge as an informed decision linked to the climate risk output.

### 4.1 Possible causes of low agreement

As indicated in the introduction section, the JRC is in charge of evaluating the adaptation plans submitted by the signatories. Due to the knowledge accumulated by the authors in the assessments carried out and feedback reports delivered so far, we have hypothesized a list of possible causes for the low agreement detected in the analysis presented in the previous section. Therefore, the following list should only be considered as hypothesis that could be explored in future research:

• There might be a lack of expertise in the person in charge of uploading the information on the *MyCovenant* platform. (For example, if the person in charge is not familiar with the concepts of hazard, exposure, vulnerability, risk, impact, and adaptation (see Table 7)), s(he) could upload misleading information in the *MyCovenant* platform. Thus, it is important to highlight that hazard level is not interchangeable with risk.
• The technicians are using different climate models and sources of information, leading to low agreement in the clusters. Global climate models have less resolution than regional climate models and they cannot resolve micro-scale processes (like deep convection) that can lead to some of the hazards included here like flash floods and storms. On the other side, regional models produced high-resolution outputs, but their calibration and bias correction depends from a dense network of observations.
• *MyCovenant* platform is confusing. There is a misuse of the concepts “hazard” and “risk” (see Table 1). MyCovenant platform refers to current hazards as “current hazard risk level”, mixing the concepts of hazard and risk (see Table 7). Hazards are only a component of the risk, along with exposure and vulnerability. Similarly, *MyCovenant* platform refers to the expected change in intensity and frequency as “anticipated risks”. The risk cannot change in intensity and/or frequency, but the hazard itself. Consequently, this might be confusing the person in charge of uploading the information. In fact, the variability could potentially tend to 1 if the risk is considered (since the variables exposure and vulnerability come into play); however, if the term used is “hazard” the variability should instead tend to 0 as mentioned above.
• Time frame of the CoM reporting platform (0–5 years, 5–15 years, and over 15 years) is not compatible with the time frames normally addressed by the models, e.g. a period centre in 2050 and 2100, making difficult to select the appropriate time frame by the Municipalities (short, medium, long-term). Therefore, the time frame may be randomly selected, since, if the logic of climate models is applied, all time frames should have been defined as long-term.
• Municipalities' leaders hide (or deny) information concerning the dangerousness of climate hazards for economic (Grasso, 2019), political (van Wijnbergen and Willems, 2015; Wishart, 2019) and/or social reasons (Bowden et al., 2019).

### 4.2 Possible solutions

According to the authors of this paper (on the basis of their accumulated knowledge after having assessed all the adaptation plans submitted so far by the CoM signatories), the previous possible causes of low agreement could be tackled through the implementation of the following actions (see also Table 6):

• Lack of expertise of the person in charge of uploading the information. The Municipality could appoint a qualified person in climate change adaptation to review and upload the information on the *MyCovenant* platform. If the Municipality is too small or does not have enough resources to allocate qualified people to this task, the figure of the CTC could emerge as an authority at higher level of governance, providing the Municipalities with strategic guidance, financial and technical support (Melica et al., 2018), being particularly useful for uploading the information on the platform.
• Use of different climate models and sources of information. Whenever possible it is desirable to use standardised or agreed information, for instance model outputs from regional initiatives like EURO-CORDEX.
• Confusing reporting platform. The Covenant of Mayors Office could change the wording of the reporting platform to refer to the agreed concepts, as defined in Table 7. Furthermore, following the comments given in section 3.3, sea level rise is a slow onset hazard that will contribute to increase the intensity and frequency of coastal flooding as long as it is associated with storm surges (Vousdoukas et al., 2017, 2018). Consequently, sea level rise should be a complementary climate hazard type along with storm surge.
• The reporting timeframe of the CoM should be better aligned with the timeframe of the models.
• Hidden information (or deny) concerning the dangerousness of climate hazards. An exercise of comparing what the Municipalities report in MyCovenant with what climate simulations provide could be enough to identify outliers. Then, further specific research might be developed to make sure the hypothetical outliers are (not) hiding information.

**Table 6:** Possible causes and solutions to the low agreement detected.

Possible cause of low agreement	Possible solution
Lack of expertise	Designate a qualified person for implementing the task
	Carry out capacity building workshops by the Covenant of Mayors Office
	Ask for help to the corresponding CTC in case of existing
Climate models' uncertainty	Use of standardised regional climate models in the CoM
Confusing reporting platform	Use IPCC definitions (as given in Table 7) in the platform. “Current hazard risk level” has to be changed by “current hazard level”. Similarly, “anticipated risks” has to be changed by “anticipated hazards”
	Merge sea level rise with storm surges in the climate hazard type column
	Re-define the timeframe using climate models' approach
Hidden information	Specific research

**Table 7:** Key concepts of climate risk and adaptation.

Concept	Definition
Hazard	The potential occurrence of a natural or human-induced physical event or trend or physical impact that may cause loss of life, injury, or other health impacts, as well as damage and loss to property, infrastructure, livelihoods, service provision, ecosystems and environmental resources.
Exposure	The presence of people, livelihoods, species or ecosystems, environmental functions, services, and resources, infrastructure, or economic, social, or cultural assets in places and settings that could be adversely affected.
Vulnerability	The propensity or predisposition to be adversely affected. Vulnerability encompasses a variety of concepts and elements including sensitivity or susceptibility to harm and lack of capacity to cope and adapt.
Risk	The potential for consequences where something of value is at stake and where the outcome is uncertain, recognizing the diversity of values. Risk is often represented as probability or likelihood of occurrence of hazardous events or trends multiplied by the impacts if these events or trends occur.
Impact	Effects on natural and human systems. [It] is used primarily to refer to the effects on natural and human systems of extreme weather and climate events and of climate change. Impacts generally refer to effects on lives, livelihoods, health, ecosystems, economies, societies, cultures, services and infrastructure due to the interaction of climate changes or hazardous climate events occurring within a specific time period and the vulnerability of an exposed society or system. Impacts are also referred to as consequences and outcomes. The impacts of climate change on geophysical systems, including floods, droughts and sea level rise, are a subset of impacts called physical impacts.
Adaptation	The process of adjustment to actual or expected climate and its effects. In human systems, adaptation seeks to moderate or avoid harm or exploit beneficial opportunities. In some natural systems, human intervention may facilitate adjustment to expected climate and its effects.

Source: IPCC (2014).

## 5 Conclusions

This paper presented, for the first time, a thorough analysis of climate hazard data of CoM signatories as well as the degree of agreement of those signatories when reporting climate data. Thus, we assumed that the signatories belonging to the same climate region have reported similar climate hazard data for both present and future. In order to answer this research question, we applied a measure of variability for categorical data.

In general, the reported data shows a low level of agreement, possibly indicating a low effectiveness of the reporting template of the CoM concerning the climate risk assessment of the adaptation pillar. As a consequence of this, we have listed possible causes of this low agreement, such as: 1) lack of expertise, 2) use of different climate models, 3) confusing reporting platforms (misuse of the concepts “hazard” and “risk, the inclusion of sea level rise as a hazard that might change in intensity and frequency, and inappropriate time frames for the future evolution of hazards), and 4) the hypothetical desire to hide or deny hazardous events by Municipalities for economic, political, social and/or cultural reasons.

The authors believe that the most likely cause of the low agreement found is the confusing reporting template, due to the fact that is the only cause that can be proved, whereas the others are only additional hypothesis that could also explain the low agreement. Notwithstanding, the authors do not discard the other causes either. As a result, and according to our analysis, the low agreement found within climate regions shows that the reporting platform would benefit from a better definition of concepts and questions. Furthermore, other solutions to improve the effectiveness of the reporting would be to raise the expertise of reporting staff and use standardized climate models.

## Declarations

### Author contribution statement

Yeray Hernandez, Gustavo Naumann: Conceived and designed the experiments; Performed the experiments; Analyzed and interpreted the data; Wrote the paper.

Paulo Barbosa: Conceived and designed the experiments; Analyzed and interpreted the data; Wrote the paper.

### Funding statement

This research did not receive any specific grant from funding agencies in the public, commercial, or not-for-profit sectors.

### Competing interest statement

The authors declare no conflict of interest.

### Additional information

No additional information is available for this paper.
